# Supplementary material for: Bioinspired Histidine–Zn2+ Coordination for Tuning the Mechanical Properties of Self-Healing Coiled Coil Cross-Linked Hydrogels
Source: Biomimetics (Basel). 2019 Mar 18;4(1):25. doi: 10.3390/biomimetics4010025 (PMC6477626; doi:10.3390/biomimetics4010025)
Supplement: Supplementary file 1 [file biomimetics-04-00025-s001.pdf]

*Biomimetics* **2019**, *4*, 25

# Supplementary Materials: Bioinspired Histidine–$Zn^{2+}$ Coordination for Tuning the Mechanical Properties of Self-Healing Coiled Coil Cross-Linked Hydrogels

**Isabell Tunn [1], Matthew J. Harrington [2,3,*] and Kerstin G. Blank [1,*]**

[1] Max Planck Institute of Colloids and Interfaces, Mechano(bio)chemistry, Science Park Potsdam-Golm, 14424 Potsdam, Germany; Isabell.Tunn@mpikg.mpg.de
[2] Max Planck Institute of Colloids and Interfaces, Department of Biomaterials, Science Park Potsdam-Golm, 14424 Potsdam, Germany
[3] McGill University, Department of Chemistry, 801 Sherbrooke St. West, Montreal, Quebec H3A 0B8, Canada
* Correspondence: Kerstin.Blank@mpikg.mpg.de (K.G.B.); Matt.Harrington@mcgill.ca (M.J.H.); Tel.: +49-331-567-9700 (K.G.B.); Tel.: +1-514-398-3180 (M.J.H.)

**Table of Contents**

1. MALDI-TOF Mass Spectra of Peptides $A_{4H3}$ and $B_{4H3}$ ………………………………………..… 2
2. CD Spectroscopy ……………………………………………………………………………... 4
3. Oscillatory Shear Rheology …………………………………………………………….…. 5

## 1. MALDI-TOF Mass Spectra of Peptides A4H3 and B4H3

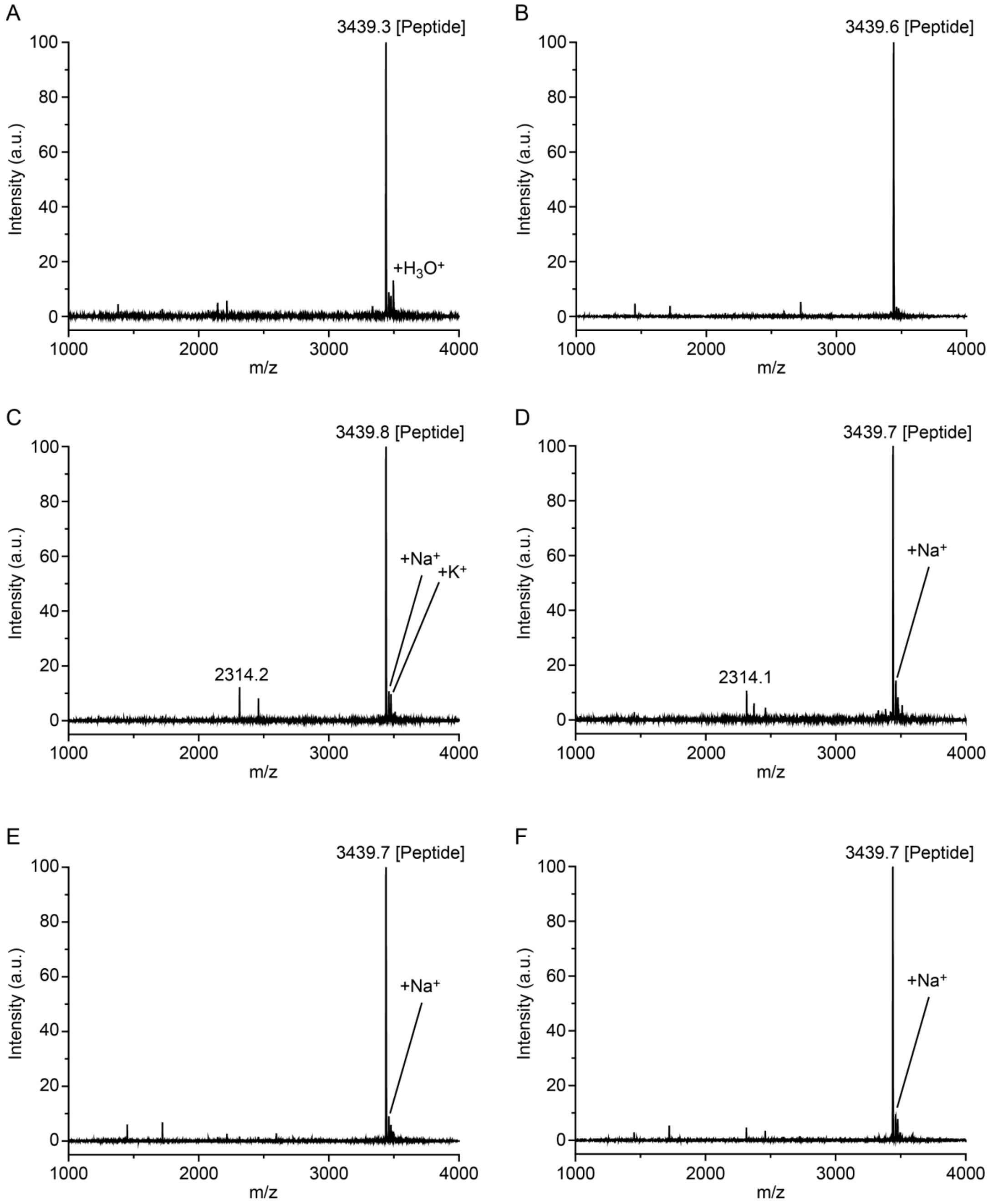


**Figure S1.** (**A**–**F**) MALDI-TOF mass spectra of the different fractions of $A_{4H3}$ used for CD and Raman spectroscopy as well as hydrogel formation. DHB was used as the matrix. The spectra were normalized from 0 to 100 (maximum intensity). The calculated mass of $A_{4H3}$ is 3437.8 g mol$^{-1}$. Sodium adduct: $Na^+$; potassium adduct: $K^+$; hydronium adduct: H3O$^+$.

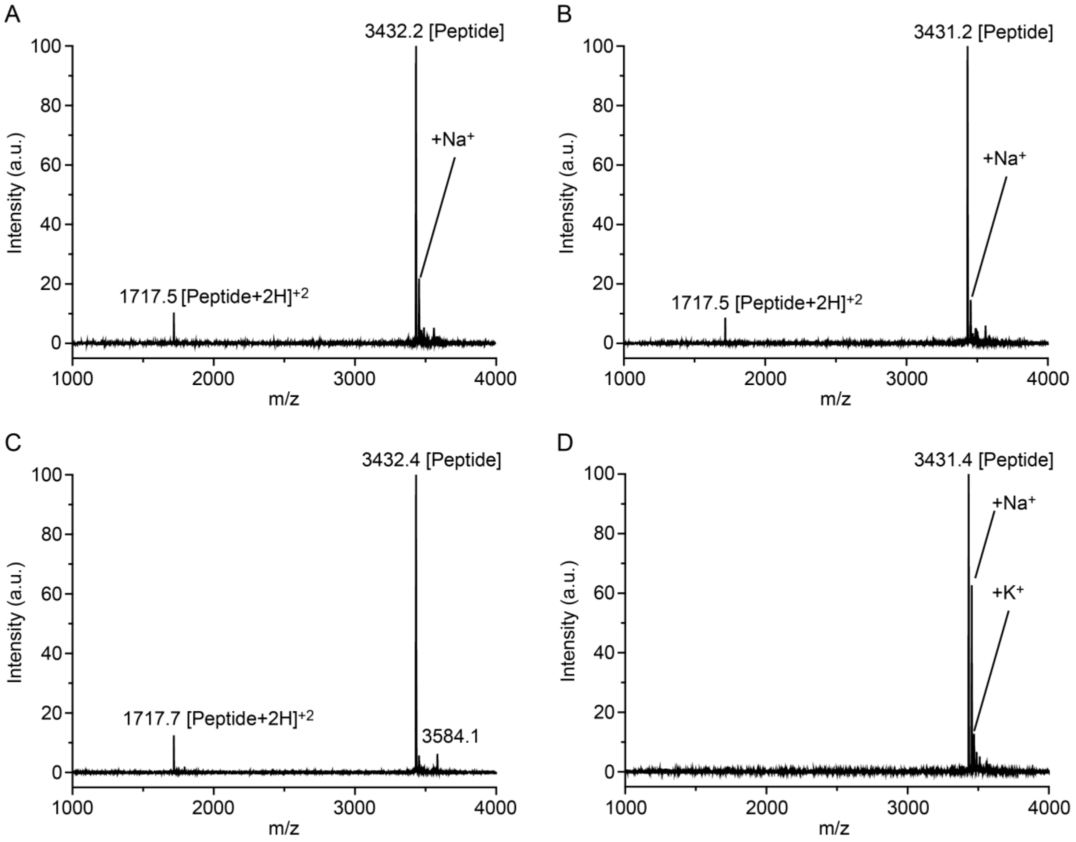


**Figure S2.** (**A**–**D**) MALDI-TOF mass spectra of the different fractions of $B_{4H3}$ used for CD and Raman spectroscopy as well as hydrogel formation. DHB was used as the matrix. The spectra were normalized from 0 to 100 (maximum intensity). The calculated mass of $B_{4H3}$ is 3430.2 g $mol^{-1}$. Sodium adduct: $Na^+$; potassium adduct: $K^+$.

## 2. CD Spectroscopy

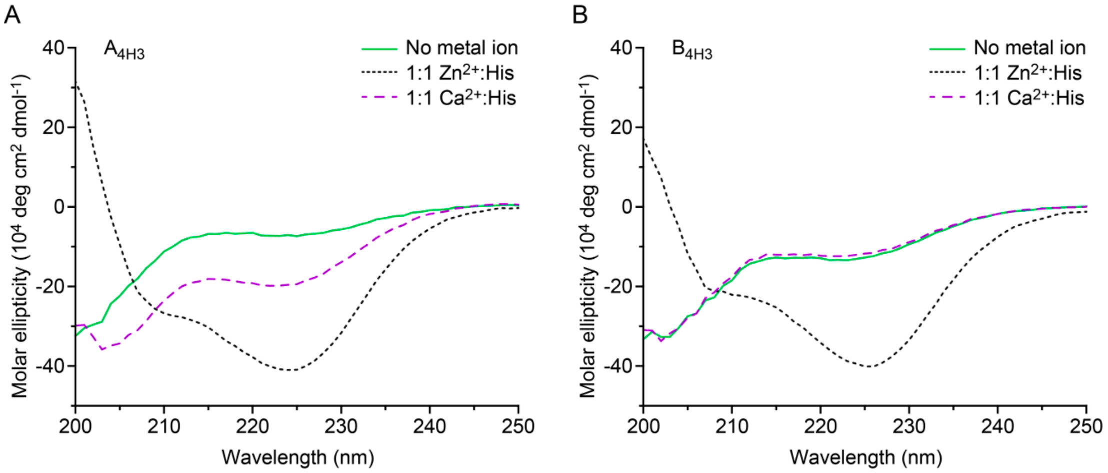


**Figure S3.** CD spectra of (**A**) $A_{4H3}$ and (**B**) $B_{4H3}$ without metal ions and in the presence of 1:1 $Zn^{2+}$:His and 1:1 $Ca^{2+}$:His. The spectra were measured at a concentration of 50 μM peptide in PIPPS buffer at room temperature.

## 3. Oscillatory Shear Rheology

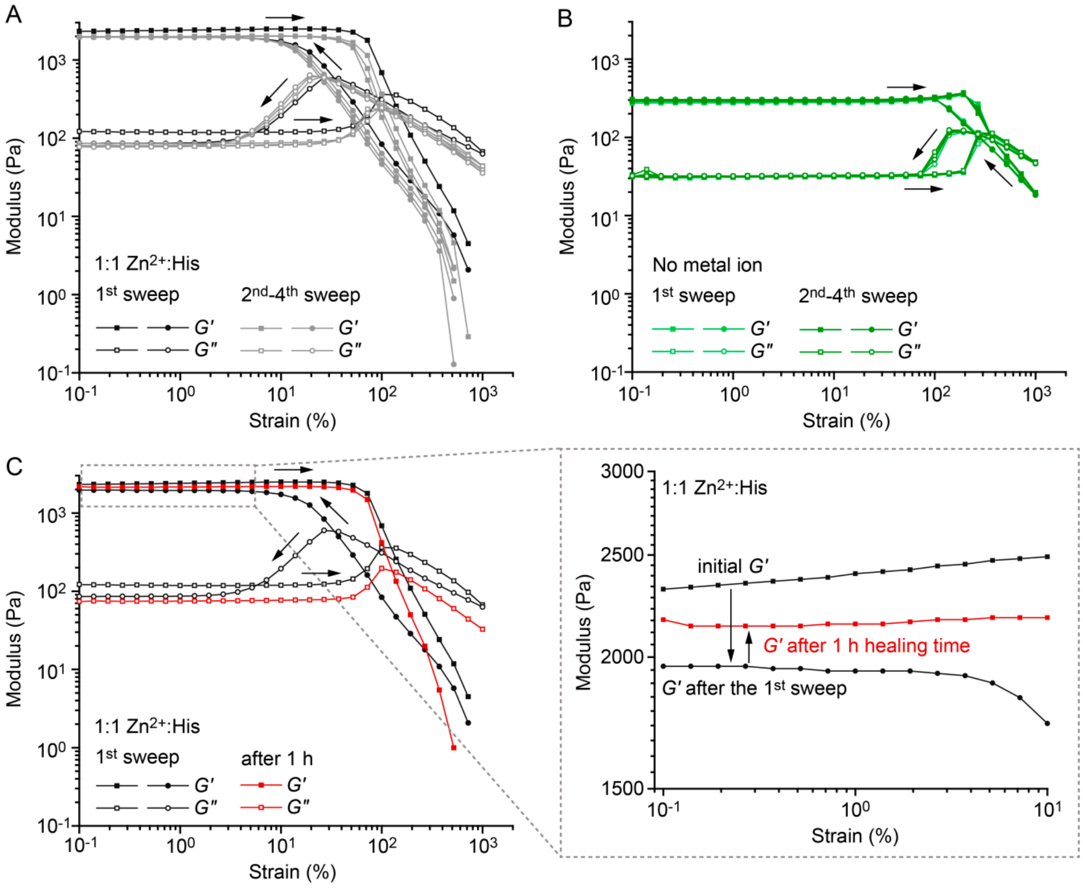


**Figure S4.** Amplitude sweeps of the $A_{4H3}B_{4H3}$-containing hydrogel in PIPPS buffer (**A**) with 1:1 $Zn^{2+}$:His and (**B**) without metal ions. Amplitude sweeps were performed at a constant angular frequency of 10 rad $s^{-1}$, changing the strain amplitude from 0.1% to 1000% and vice versa. After the first amplitude sweep the hydrogel only recovered ≈80% of *G*′. The measurement was repeated four times without a further decrease in *G*′. (**C**) To investigate the self-healing behavior of the $Zn^{2+}$-fortified hydrogel, the hydrogel was allowed to rest for 1 h after four amplitude sweeps. Then, an additional amplitude sweep was performed and revealed the recovery of *G*′ to ≈90% of the initial value. Inset: Zoom into the linear viscoelastic region of *G*′ of the $Zn^{2+}$-fortified hydrogel.

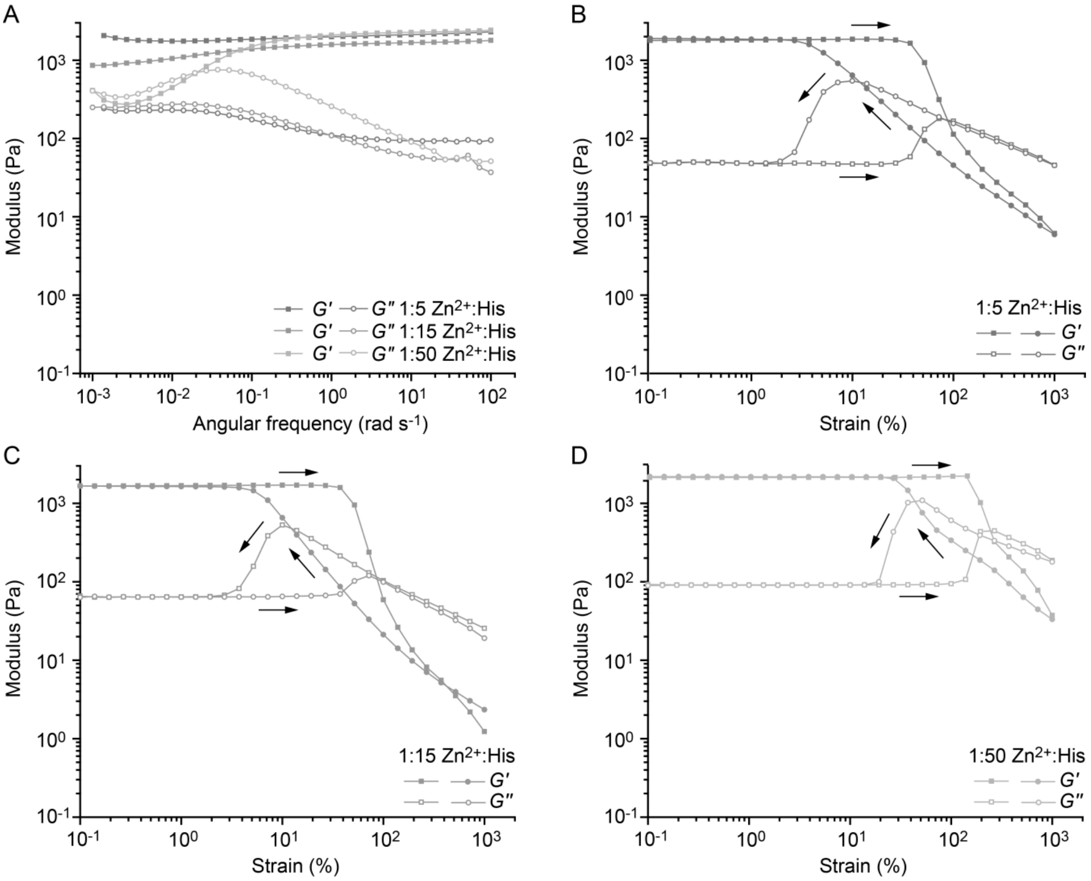


**Figure S5.** Frequency and amplitude sweeps of $A_{4H3}B_{4H3}$-containing hydrogels in PIPPS buffer with different $Zn^{2+}$:His ratios. (**A**) Frequency sweeps were performed from 100 to 0.001 rad $s^{-1}$ at a constant strain amplitude of 1%. Amplitude sweeps in the presence of (**B**) 1:5, (**C**) 1:15 and (**D**) 1:50 $Zn^{2+}$:His were performed at a constant angular frequency of 10 rad $s^{-1}$, changing the strain amplitude from 0.1% to 1000% and vice versa .

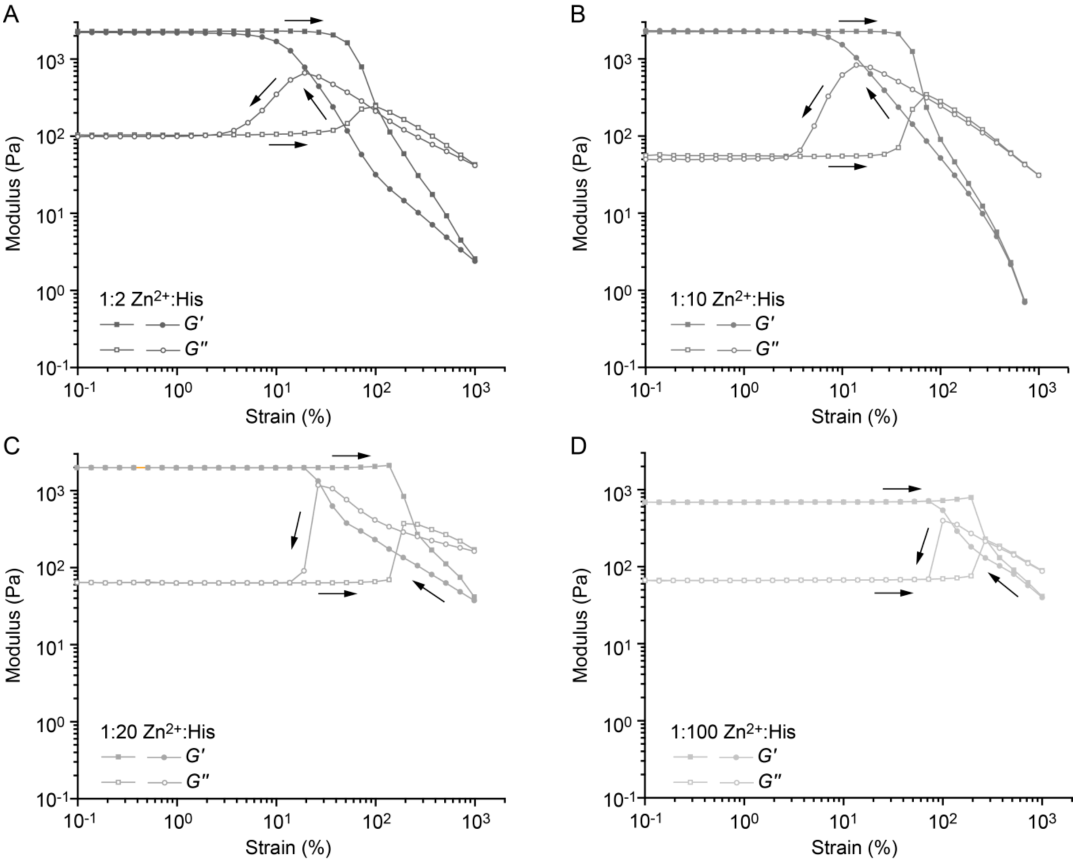


**Figure S6.** Amplitude sweeps of $A_{4H3}B_{4H3}$-containing hydrogels in PIPPS buffer with different $Zn^{2+}$:His ratios. Amplitude sweeps in the presence of (**A**) 1:2, (**B**) 1:10, (**C**) 1:20 and (**D**) 1:100 $Zn^{2+}$:His were performed at a constant angular frequency of 10 rad $s^{-1}$, changing the strain amplitude from 0.1% to 1000% and vice versa.

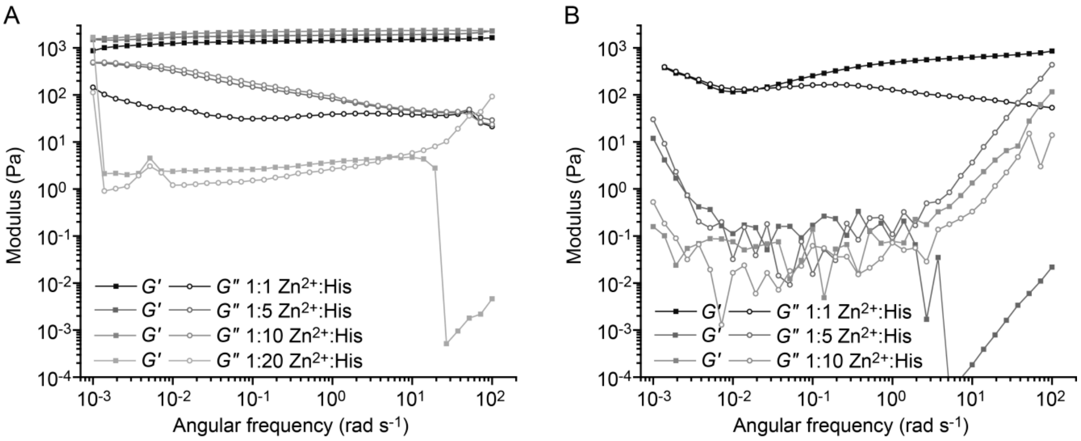


**Figure S7.** Frequency sweeps of the individual star-PEG–peptide conjugates (**A**) star-PEG–$A_{4H3}$ and (**B**) star-PEG–$B_{4H3}$. The measurements were performed in PIPPS buffer, using $Zn^{2+}$:His ratios down to 1:20 (**A**) or down to 1:10 (**B**). Frequency sweeps were performed from 100 to 0.001 rad $s^{-1}$ at a constant strain amplitude of 1%.

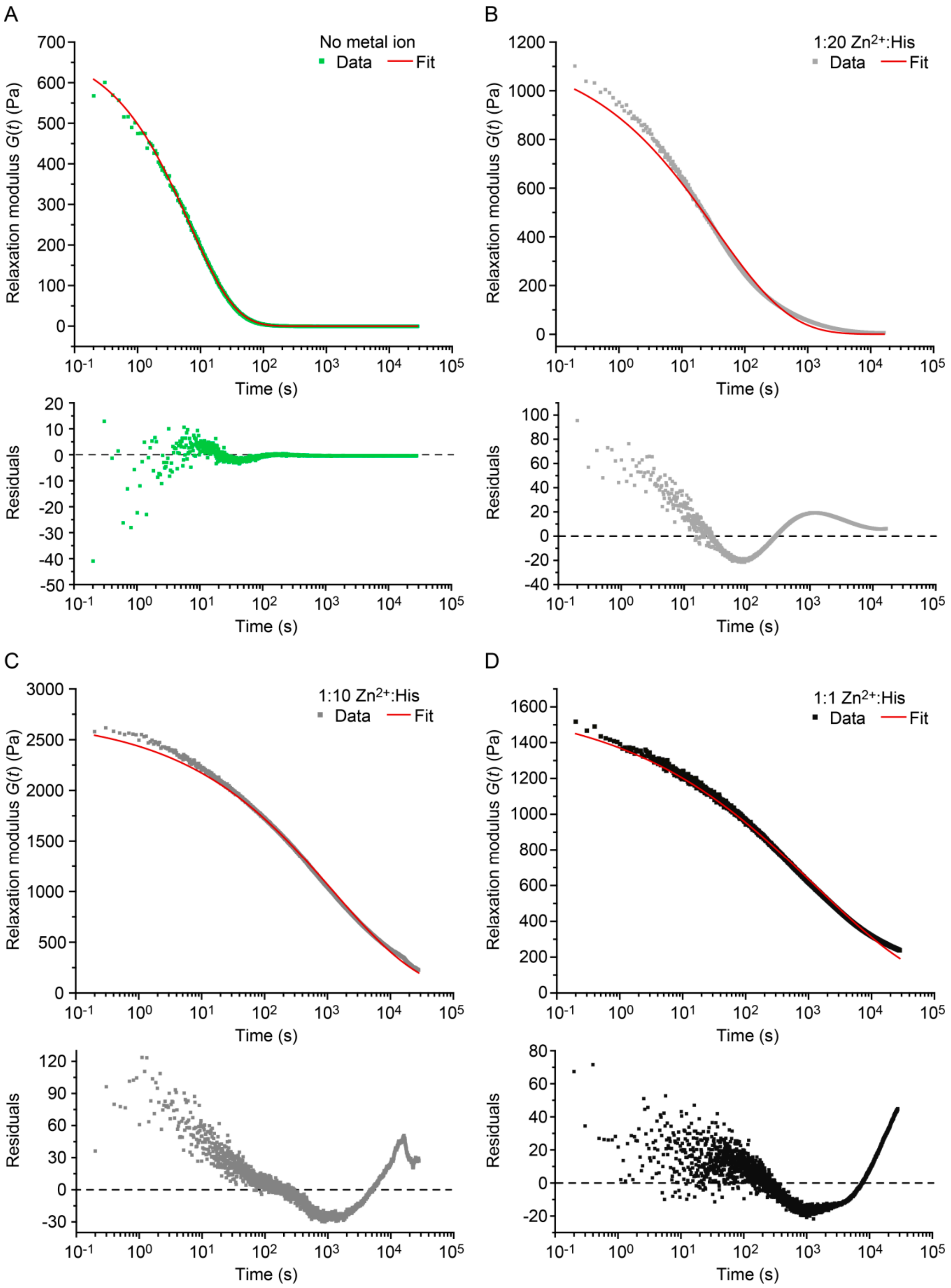


**Figure S8.** Stress relaxation of $A_{4H3}B_{4H3}$-containing hydrogels in PIPPS buffer (**A**) without metal ions, with (**B**) 1:1, (**C**) 1:10 and (**D**) 1:20 $Zn^{2+}$:His. The stress relaxation of the hydrogels was monitored after applying a step strain of 10%. The relaxation modulus $G(t)$ was fitted to Kohlrausch's stretched exponential relaxation model to obtain the relaxation time $\tau_{sr}$ (Supplementary Equation (S1)). The fit parameters can be found in Supplementary Table S2. The fit residuals are shown below the fit of the corresponding relaxation data.

The fit of the stress relaxation data (Supplementary Figure S8) with the Kohlrausch's stretched exponential relaxation model (Supplementary Equation (S1)) was performed with the Levenberg–Marquardt iteration mechanism in Origin Pro 2015 b9.2.257.

$$G(t) = G_0\, exp(-(t/\tau_{sr})^{\alpha}) \qquad \text{(S1)}$$

The relaxation time $\tau_{sr}$ and the fitting parameter $\alpha$ are reported in Supplementary Table S2. The initial plateau modulus $G_0$ was fixed to the value of $G'$ in the linear viscoelastic range, obtained from amplitude sweeps of the same sample performed before the stress relaxation experiment.

**Table S1.** Relaxation times $\tau_{fs}$ obtained from frequency sweeps of the $A_{4H3}B_{4H3}$-containing hydrogels in PIPPS buffer. [1]

| $A_{4H3}B_{4H3}$ | No metal ion | 1:1 $Zn^{2+}$:His | 1:1 $Ca^{2+}$:His | 1:1 $Zn^{2+}$:His + EDTA |
|---|---|---|---|---|
| $\tau$fs, 1st repeat (s) | 7.2 | >1000 | 5.2 | 5.2 |
| $\tau$fs, 2nd repeat (s) | 7.2 | >1000 | 5.2 | 7.2 |
| $\tau$fs, 3rd repeat (s) | 19.3 | >1000 | 3.7 | 5.2 |
| Mean ± SEM (s) | 11.2 ± 4.0 | - | 4.7 ± 0.5 | 5.9 ± 0.7 |

[1] Frequency sweeps were performed from 100 to 0.001 rad $s^{-1}$ at a constant strain amplitude of 1%. The mean and the standard error of mean (SEM) were calculated ($n$ = 3).

**Table S2.** Relaxation time of the $A_{4H3}B_{4H3}$-containing hydrogels at different $Zn^{2+}$:His ratios obtained from the crossover of $G'$ and $G''$ in the frequency sweeps ($\tau_{fs}$) or stress relaxation experiments ($\tau_{sr}$). [1]

| **Hydrogel** | **$\tau_{fs}$ (s)** | **$\tau_{sr}$ (s)** | **$\alpha$** | **$G_0$ (Pa)** |
|---|---|---|---|---|
| No metal ion | 11.2 | 6.6 | 0.59 | 690 |
| 1:20 $Zn^{2+}$:His | 51.8 | 34.4 | 0.37 | 1170 |
| 1:10 $Zn^{2+}$:His | >1000 | 1244.0 | 0.31 | 2720 |
| 1:1 $Zn^{2+}$:His | >1000 | 1272.0 | 0.24 | 1630 |

[1] The stress relaxation curves were fitted to Kohlrausch's stretched exponential relaxation model to obtain the relaxation time $\tau_{sr}$. $G_0$ is the initial plateau modulus and $\alpha$ the fitting parameter resulting from physical constraints of the cross-links in the material. $G_0$ was fixed to the value of $G'$ in the linear viscoelastic region of the amplitude sweep of the same hydrogel performed before the relaxation experiment.
